# Ecology of the gut microbiota and colonization resistance: mechanisms and therapeutic implications

**DOI:** 10.1093/femsec/fiaf124

**Published:** 2025-12-09

**Authors:** Lanfan Liang, Ziyi Yang, Xiangsheng Fu

**Affiliations:** Department of Gastroenterology, Clinical Medical College and the First Affiliated Hospital of Chengdu Medical College, Chengdu, Sichuan 610500, P. R. China; Key Laboratory of Digestive Microecology Medicine of Sichuan Health Commission, Chengdu, Sichuan 610500, P. R. China; Key Laboratory of Digestive System Tumors and Microenvironment of Sichuan Provincial University, Chengdu, Sichuan 610500, P. R. China; Department of Gastroenterology, Clinical Medical College and the First Affiliated Hospital of Chengdu Medical College, Chengdu, Sichuan 610500, P. R. China; Key Laboratory of Digestive Microecology Medicine of Sichuan Health Commission, Chengdu, Sichuan 610500, P. R. China; Key Laboratory of Digestive System Tumors and Microenvironment of Sichuan Provincial University, Chengdu, Sichuan 610500, P. R. China; Department of Gastroenterology, Clinical Medical College and the First Affiliated Hospital of Chengdu Medical College, Chengdu, Sichuan 610500, P. R. China; Key Laboratory of Digestive Microecology Medicine of Sichuan Health Commission, Chengdu, Sichuan 610500, P. R. China; Key Laboratory of Digestive System Tumors and Microenvironment of Sichuan Provincial University, Chengdu, Sichuan 610500, P. R. China

**Keywords:** gut microbiota, ecology, host, colonization resistance, therapeutic implications

## Abstract

The human gastrointestinal (GI) ecosystem is a highly dynamic environment and provides diverse microbial habitats for the gut microbiota, which are shaped by environmental factors, metabolic processes, and immune responses. The host–microbiota interactions in the gut form a balanced yet adaptable network. When invading microorganisms enter the GI tract, they deploy multiple strategies to overcome both host defences and competition from the resident microbiota. In turn, the host and native microbiota have evolved sophisticated mechanisms to prevent the colonization of invading organisms, collectively termed colonization resistance. Deciphering the mechanisms of interplay in the host‒microbe and microbe‒microbe relationships in the gut offers crucial insights into therapeutic interventions aimed at restoring or maintaining gut microbial homeostasis.

## Teaser

This MiniReview explores how the gut’s native microbes and the host jointly prevent foreign invaders from colonizing, a defense mechanism known as colonization resistance, with crucial therapeutic implications.

## Introduction

**Figure 1.**
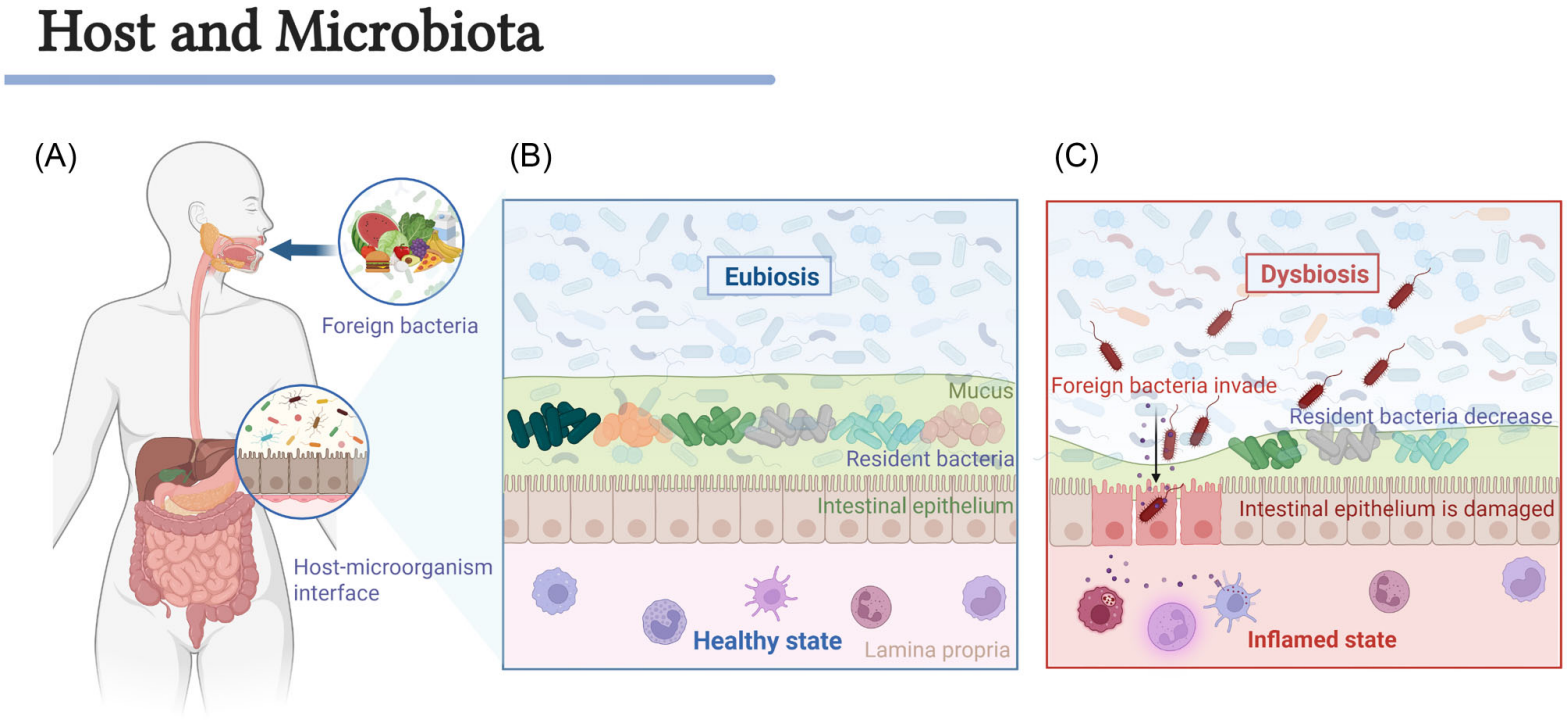
The complex interplay between foreign microbes, resident microbiota, and the host determines gut microbial homeostasis or dysbiosis. (A) Foreign microorganisms enter the gut through the oral route and interact with commensal microbiota. (B) Microbial communities form stable symbiotic relationships with their hosts. (C) Colonization of foreign microbes disrupts the barrier function of the GI tract mucosa, leading to dysbiosis and inflammation. Created with BioRender.com.

The gut harbours a diverse ecosystem of trillions of coevolved microorganisms, including bacteria, viruses, fungi, archaea, and protozoa, which maintain mutually balanced relationships with their host organism (Wang et al. 2016, Caballero-Flores et al. 2023). The human gastrointestinal (GI) ecosystem, with its vast surface area (∼400 m²) (Peterson and Artis 2014), is highly dynamic and provides diverse microbial habitats (Bäckhed et al. 2005). In specific intestinal niches, certain microbial taxa establish themselves as permanent residents, whereas others act as transient hitchhikers from ingested food, water, and environmental components (Gutiérrez-García et al. 2024) (Fig. 1A).

Through long-term evolution, indigenous microbial communities in the gut have formed stable symbiotic relationships with their hosts (Fig. 1B), while ingested invading microbes can colonize gut ecological networks through complex interactions with resident symbionts (Gutiérrez-García et al. 2024). However, excessive colonization by invading microbes may disrupt the barrier function of the GI tract mucosa, leading to dysbiosis and intestinal inflammation (Fig. 1C). Thus, invading microbes encounter colonization resistance, a mechanism by which the host and commensal microbiota collectively block microbial invasion into local communities through multilayered barriers (Spragge et al. 2023). These interactions reflect the specificity of natural selection and further provide evidence for multilevel ecological networks in the gut (Gillingham et al. 2025).

Owing to the diversity and complexity of the gut microbiota, in this review, we focus on bacteria as the main subject of discussion because of their key role in gut microbial ecology and previously well-characterized mechanisms.

## The microenvironment of the gut determines the spatial distribution of bacteria

**Figure 2.**
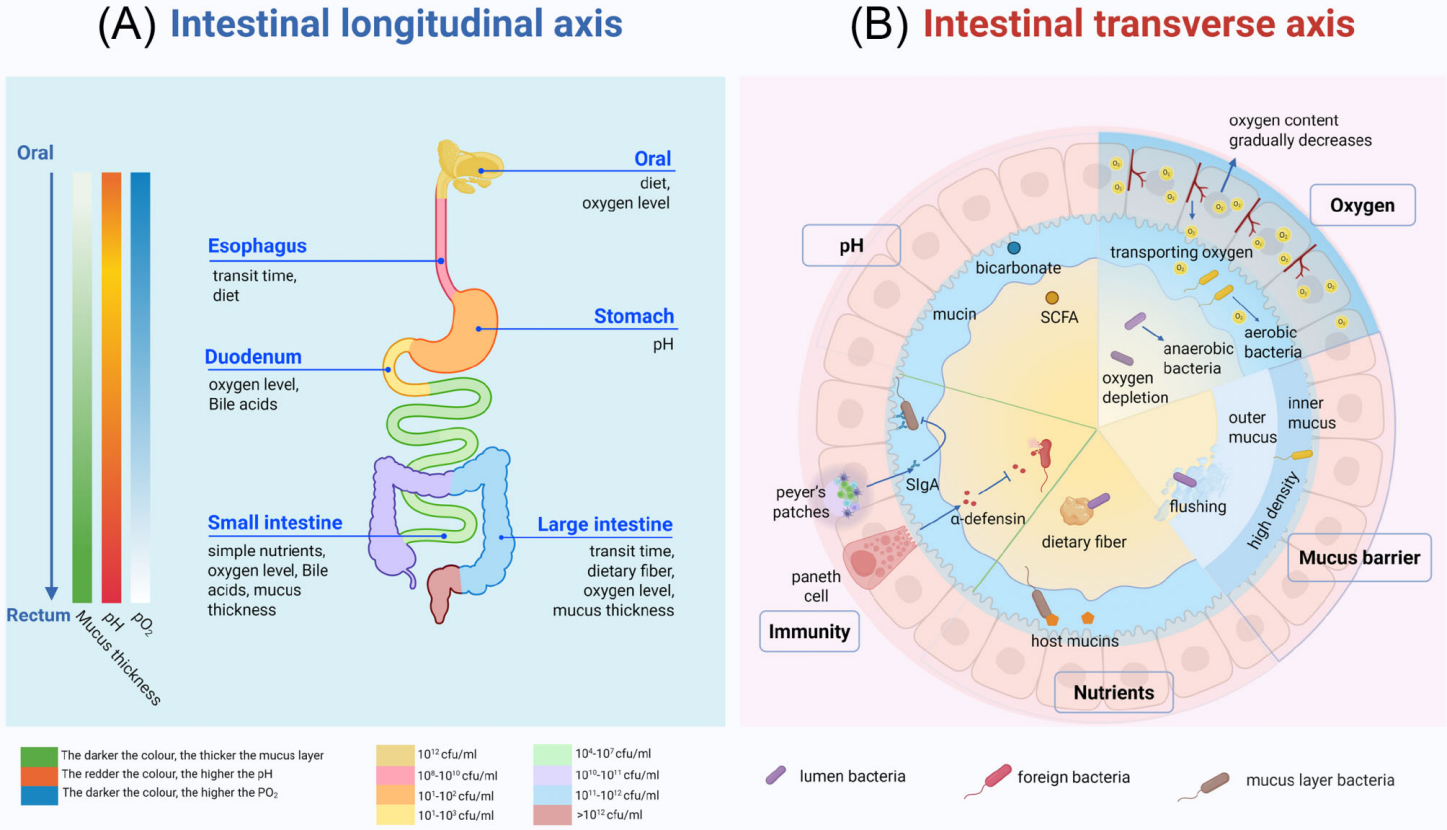
Heterogeneous distribution of gut microbiota along the longitudinal and vertical axes and its determining factors. (A) The schematic illustrates key physicochemical parameter changes (oxygen partial pressure, pH, bile acid concentration, and luminal flow rate) along the gastrointestinal tract from the proximal to distal regions, alongside the corresponding shifts in dominant bacterial taxa. (B) The cross-sectional schematic reveals differences in oxygen tension, pH, nutrient sources, and host immune factors across the three primary niches of the lumen, mucus layer, and mucosal epithelium, which collectively shape distinct microbial communities in each niche. Created with BioRender.com.

The abundance and diversity of the gut microbiota tend to increase from the proximal to distal regions of the gut (Martinez-Guryn et al. 2019). This spatial heterogeneity arises from regional differences along the longitudinal and transverse axes of the gut due to a variety of factors, including pH, oxygen tension, nutrient composition, bile acid concentration, lumen transport rate, mucosal characteristics, and immune factors (Tropini et al. 2017, Martinez-Guryn et al. 2019) (Fig. 2).

On the longitudinal axis of the GI tract, oxygen levels decrease gradually from the stomach to the distal sigmoid colon, resulting in a difference in the distribution between aerobic and anaerobic bacteria. The measured oxygen partial pressure in mice has been shown to decrease from 58 mmHg in the stomach to 3 mmHg near the distal sigmoid colon (Albenberg et al. 2014). *Enterococcus* and *Lactobacillus* dominate the oxygen-rich small intestine, whereas *Bacillota* and *Bacteroidota* primarily thrive in the anaerobic large intestine (Liévin-Le Moal and Servin 2006, Tropini et al. 2017). The pH gradient (from gastric pH 2–3.5 (Quigley and Turnberg 1987, Schreiber et al. 2004) to distal colon pH 6.5–7.0 (Evans et al. 1988)) further shapes microbial communities. The acidic environment of the stomach restricts invading bacterial survival, whereas the near-neutral pH of the colon increases microbial diversity (Martinez-Guryn et al. 2019). In the small intestine, rapid transit and abundant simple nutrients favour fast-growing facultative anaerobes such as *Proteobacteria* and *Lactobacillus* (Zmora et al. 2018). In contrast, the slower flow in the large intestine and the presence of undigested fibre favour bacteria dominated by saccharolytic *Bacteroidales* and *Bacillota* (Tropini et al. 2017). Diet (Ross et al. 2024) and bile acids (Sannasiddappa et al. 2017) also affect microbial composition. Host-derived bile acids limit bacterial growth in the small intestine, keeping microbial density in the proximal region at a relatively low level (Begley et al. 2005) (Fig. 2A) (Table 1).

**Table 1.** Biogeographical features across the longitudinal axis of the digestive tract.

Site	PH	Oxygen (mmHg)	Immune	Wriggle	Mucus barrier (μm)	Digestive fluid	Nutrient	Characteristic flora	Reference
Oral cavity	6.5–7.0	140–160	sIgA, lysozyme	∼1 min	Unknow	Saliva	Fructose	Streptococcus, Veillonella	de Vos et al. (2022)
Esophagus	6.5–7.5	40–100	MALT	∼1 min	Unknow	Unknow	Fermentable carbohydrates	Streptococcus	de Vos et al. (2022)
Stomach	1.5–3.5	58	pepsin	1–6 h	178–315	Gastric acid	Amino acids	*Helicobacter pylori*	de Vos et al. (2022), Groisman et al. (2023), She et al. (2024)
Duodenum	4.0–6.0	30–60	Peyer’s Patches	2–15 h	∼500	Primary bile acids	Lipid	Actinobacteria, Aspergillus	Rivera-Chávez et al. (2016), Zmora et al. (2019), Duller et al. (2024), Ross et al. (2024), She et al. (2024)
Jejunum	6.0–7.4	10–34	Paneth cell	2–15 h	119–127	Primary bile acids	Carbohydrates, proteins	Firmicutes	Rivera-Chávez et al. (2016), Duller et al. (2024), Ross et al. (2024), She et al. (2024)
Ileum	7.0–8.0	<40	Paneth cell; RORγt+ T cells	2–15 h	433–527	Pancreatic fluid	B vitamins	Lactobacillaceae, Proteobacteria	Rivera-Chávez et al. (2016), de Vos et al. (2022), Duller et al. (2024), Ross et al. (2024), She et al. (2024)
Colon	6.0–7.5	3	goblet cell	3–48 h	720–940	Mucus	SCFAs	Firmicutes	Liévin-Le Moal and Servin (2006), Groisman et al. (2023), Ross et al. (2024), She et al. (2024)

The intestinal mucosa, mucus layer, and intestinal lumen differ in their cross-axis bacterial distributions because of their unique environmental characteristics, including oxygen content, pH, immune factors, nutrient composition, and mucus thickness (Albenberg et al. 2014, Tropini et al. 2017, Juge 2022). Along the transverse axis of the intestinal lumen, the mucus layer plays a crucial role in bacterial differentiation. The sterile inner mucus layer in the colon forms a physical barrier, whereas the outer loose mucus layer provides space for bacterial survival and is a primary source of nutrients derived from host mucins. The heterogeneous distribution of nutrients in the lumen drives community structure and clustering, wherein luminal-dominant bacteria such as *Bacteroides* spp. break down dietary fibre via distinct polysaccharide utilization loci-encoded lyases (La Rosa et al. 2022), while bacteria such as *Akkermansia muciniphila* exploit colonic mucus for nutrients (Johansson et al. 2011). The lumen maintains a more acidic environment because of fibre fermentation and the production of short-chain fatty acids (SCFAs). In contrast, the mucus layer and mucosal surface maintain a near-neutral pH through active bicarbonate secretion and ion buffering (Juge 2022), creating a distinct pH gradient across the transverse axis. The increasing viscosity of mucus from the proximal to distal regions also affects bacterial adhesion (McCallum and Tropini 2024). Additionally, the mucosal layer adjacent to the lamina propria maintains an optimal aerobic microenvironment (oxygen partial pressure of ∼1–10 mmHg) (Espey 2013) and is rich in epithelium-derived IgA and antimicrobial peptides, which selectively regulate bacterial growth and colonization (Tropini et al. 2017) (Fig. 2B) (Table 2).

**Table 2.** Biogeographical features across the transverse axis of the digestive tract

Site	PH	Oxygen (mmHg)	Immune	Physical barrier (colon, μm)	Metabolite	Nutrient	Characteristic flora	Reference
Mucous layer	6.8–7.4	40–50	IgA, macrophages, DC cells, T cells, B cells, cytokines	NA	SCFAs, vitamin K, B vitamins	Amino acids, glucose, fatty acids, vitamins	Actinobacteria, Proteobacteria	Groisman et al. (2023)
Inner mucus layer	6.0–6.5	5–10	IgA, anti-peptide, lysozyme	65–167	SCFAs	SCFAs, amino acids, carbohydrates, vitamins	*Bacteroides fragilis, Acinetobacter* spp	She et al. (2024)
Outer mucus layer	6.5–7.0	10–20	IgA, anti-peptide, lysozyme	605–823	Butyric acid, lactic acid, hydrogen, methane	SCFAs, bicarbonates, amino acids	Firmicutes; *Akkermansia muciniphila*	Rivera-Chávez et al. (2016), She et al.(2024)
Luminal	5.5–7.0	≤1	IgA, anti-peptides, lysozyme, complement	NA	SCFAs, lactic acid, hydrogen, methane, phenolic compounds	Carbohydrates, proteins, fats, vitamins, minerals, dietary fiber	Prevotellaceae, Bacteroidaceae	Rivera-Chávez et al. (2016), Groisman et al. (2023), Duller et al. (2024)

## Interactions in the gut microbiota: a dynamic balance of cooperation and competition

**Figure 3.**
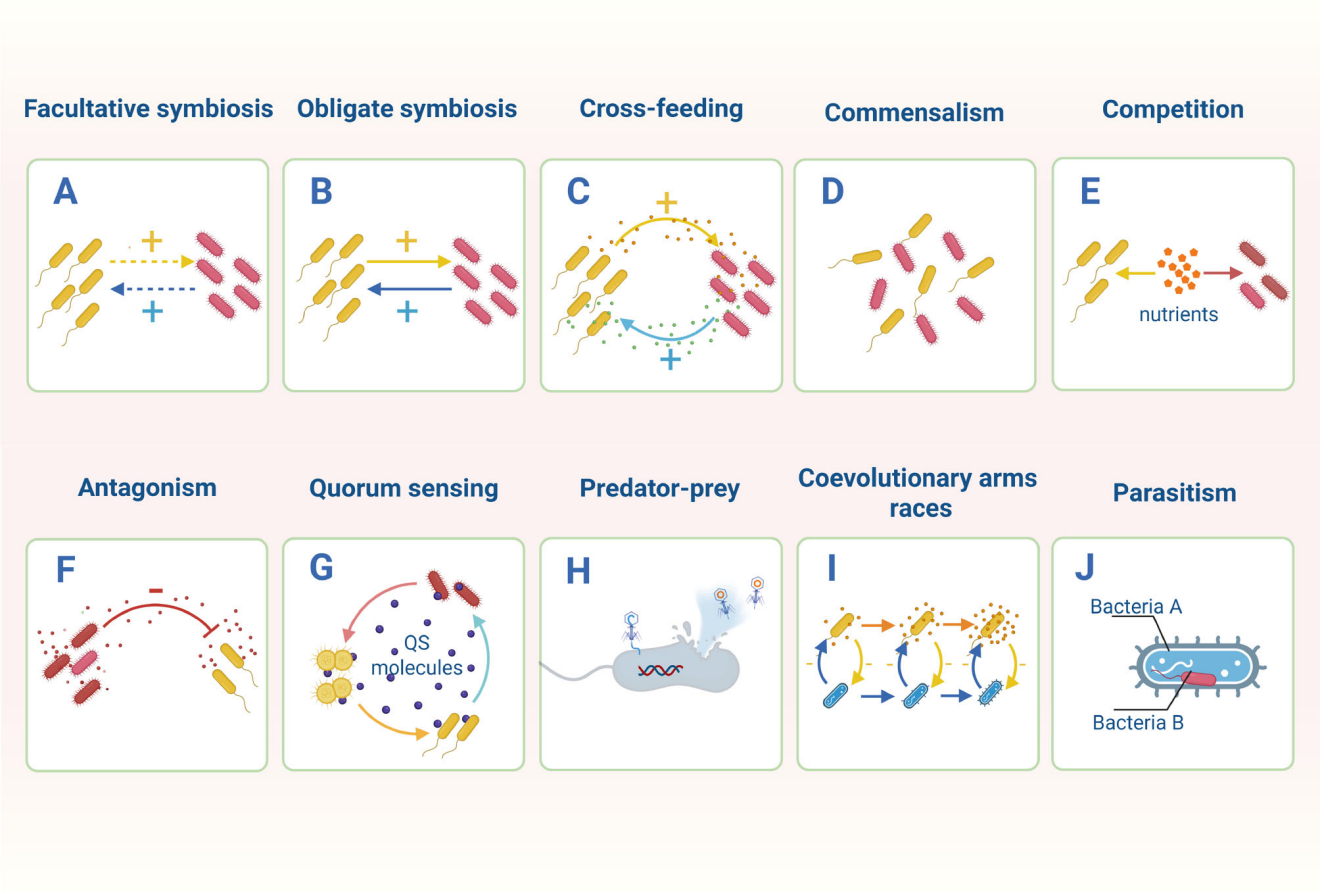
Types of interactions within gut microbiota. (A) Facultative symbiosis: microbial partners derive mutual benefits but can also survive independently under permissive conditions. (B) Obligate symbiosis: each partner depends on the other for survival and reproduction. (C) Cross-feeding: a metabolic interaction where microbes exchange nutrients. (D) Commensalism: the association between different species is beneficial to individuals of one species but has no effect on the other. (E) Competition: all partners are disadvantaged by the presence of others. (F) Antagonism: the association between different species is detrimental to individuals of one species but not to those of the other. (G) Quorum sensing: a density-dependent communication mechanism in bacteria. (H) Predator‒prey: one organism feeds on the other organism. (I) Coevolutionary arms races: recurrent cycles of adaptation and counter-adaptation between interacting bacterial lineages. (J) Parasitism: one microorganism benefits at the expense of their host organism. Created with BioRender.com.

The interactions between bacterial species within the gut microbiota are key determinants of the community’s diversity and stability, and they adopt a variety of ecological strategies to optimize colonization and survival (Rimal et al. 2024) (Fig. 3). Understanding these interconnected regulatory mechanisms provides important insights into gut microbiota interactions and evolution.

## Facultative symbiosis

Facultative symbiosis is characterized by a relatively weak mutually beneficial relationship between microbial species with optional cooperation. In such symbionts, microorganisms interact by sharing resources, coordinating metabolic activities, or exchanging genetic information (such as through horizontal gene transfer) (Lo et al. 2016), sometimes promoting the development of new morphological characteristics (Kuo and Ochman 2009). In the intestinal environment, the relationship between *Escherichia coli* (*E. coli*) and *Shigella* dynamically adjusts according to resource conditions. When resources are limited, they compete for carbohydrates, amino acids, and adhesion sites (Fischbach 2018). However, in a stable environment with abundant nutrients, they may form a symbiotic relationship. The SCFAs produced by the metabolism of *E. coli* can be utilized by *Shigella*, and the metabolic activities of *Shigella* (such as regulating the pH value) may improve the local environment and promote the survival of *E. coli* (Kamada et al. 2012, Pacheco et al. 2012) (Fig. 3A).

## Obligate symbiosis

Obligate symbiosis is characterized by a high level of interdependence between the participating organisms, with their survival or reproduction being contingent upon the relationship. This type of symbiosis often results from long-term evolution and leads to highly specialized adaptations involving metabolic, physiological, or behavioural traits that facilitate the exchange of essential resources or services between symbiotic partners (Tsoi et al. 2018). As a result, each organism that participates in this compulsory symbiosis may lose its ability to live independently outside the symbiont (McCutcheon and Moran 2011). Obligatory symbiosis may limit the adaptability of organisms to environmental changes, and the survival of the whole symbiotic system may be limited by individuals with the weakest adaptability (Pauli et al. 2022) (Fig. 3B).

## Cross-feeding

Cross-feeding, also known as metabolic symbiosis, refers to the phenomenon in which different species within microbial communities promote each other’s growth by sharing metabolites. For example, *Coryneform* bacteria cannot decompose starch by themselves, while *Polymorphous* bacteria can decompose starch through a process mediated by polysaccharide utilization sites, releasing maltose and glucose that can be used by *Coryneform* bacteria (Zafar and Saier 2021). In addition, a lack of nutrition can promote cooperation and cross-breeding between species and increase the stability of microbial communities. For example, when carbon sources are limited, *Bacteroides* can degrade complex polysaccharides to produce short-chain oligosaccharides for use by *Prevotella* (Heinken et al. 2013) (Fig. 3C).

## Commensalism

A commensal relationship among gut microflora refers to the coexistence of different microbial species within the same ecological environment. Unlike symbiosis, commensalism does not involve the direct exchange of materials or energy between organisms. In contrast, the commensal relationship may only require species to share a certain degree of space or ecological niches (Chang et al. 2023). This relationship can be expressed as the physiological activity of one microbial species, creating favourable conditions for other microbial species not directly involved in the process. For example, in the intestinal environment, the coexistence of aerobic bacteria and facultative anaerobic bacteria is beneficial for the consumption of oxygen and creation of more suitable growth conditions for anaerobic bacteria (Favier et al. 2002). In the oral cavity, *Fusobacterium nucleatum* can increase the growth of *Porphyromonas gingivalis* by establishing hypoxic habitats and producing ammonia (Kolenbrander et al. 2010). The establishment of a complex network of commensal microorganisms within the gastrointestinal microbiota usually helps to increase the diversity of gastrointestinal microorganisms (Morlon 2012) (Fig. 3D).

## Competition

Competition among microorganisms involves competition for nutritional resources and living space. Usually, in an environment with limited resources, those populations that show high affinity for specific nutrients exhibit a clear competitive advantage when nutrients are scarce. For example, *E. coli* can inhibit the growth of *Shigella* by preferentially utilizing carbon sources, and *Bacteroides* inhibits the growth of other polysaccharide-dependent microorganisms by decomposing polysaccharides in the intestine (Zafar et al. 2021). Competitive relationships play important roles in maintaining the stability of intestinal microflora, effectively preventing the proliferation and establishment of potential pathogens, and act as a natural defence against diseases (Rastall et al. 2005) (Fig. 3E).

## Antagonism

Antagonism relies on specific substances or environmental factors to prevent or limit the growth of other bacteria rather than simply competing for resources. Bacteria can relieve competitive pressure and gain advantages by producing metabolites that can destroy or kill competitors, such as antibacterial peptides and antibiotics (Riley and Wertz 2002). For example, *Lactobacillus* can effectively inhibit the growth of *Clostridium difficile* through the production of lactic acid (Rea et al. 2007). Hydrogen peroxide secreted by *Bacillus subtilis* NC533 can effectively kill *Salmonella* in vitro (Pridmore et al. 2008) (Fig. 3F).

## Quorum sensing

Communication is the core of any social structure, as is the case for bacterial communities; communication among bacteria includes both intraspecies and interspecies interactions. Bacteria use a communication system called quorum sensing, which depends on cell density, to perceive the density of surrounding cells, propagation dynamics, and species composition in their immediate environment (Laganenka et al. 2023). This system can activate or inhibit the growth, metabolic activity, or gene expression of other members of the microbial community to facilitate signal transmission and cooperation among groups (Sztajer et al. 2014) (Fig. 3G).

## Predator‒prey interactions

In the intestinal microbial community, interactions between predators and prey are crucial for maintaining balance within communities. By controlling prey populations, predators not only affect the composition and growth ability of microbial communities but also affect their diversity and stability (Reyes et al. 2010). Additionally, predation pressure may promote the evolution of microbial populations and stimulate prey to develop defence mechanisms and predators to evolve more efficient predation strategies (Muñoz et al. 2020). For example, phage therapy exploits the predation relationship between phages and bacteria to adjust the balance between symbiotic and pathogenic bacteria (Mills et al. 2013) (Fig. 3H).

## Coevolutionary arms races

Two species can evolve together and alter their survival strategies based on each other’s behaviours; this phenomenon is known as a coevolutionary arms race. During this process, species may develop new metabolic pathways, optimize offensive and defensive strategies, and regulate competition and cooperation among species (Scanlan 2017). In predator‒prey relationships, prey species gradually evolve complex defence mechanisms, such as gene editing through the CRISPR Cas system (van Houte et al. 2016), whereas predators may refine their attack strategies by utilizing anti-CRISPR-Cas systems (Borges et al. 2018) (Fig. 3I).

## Parasitism

Parasitic relationships between gut microbiota involve the utilization of one gut microbiota by another, through direct or metabolic contact, to harm another microbiota for its own benefit. Bacteriophages utilize the metabolic and replication mechanisms of bacteria to execute their own life cycles. In this process, bacteriophages obtain the resources and space necessary for reproduction, whereas host bacteria die because of damage caused by phage replication (Howard-Varona et al. 2017) (Fig. 3J).

## Strategies for invading bacterial colonization in the GI tract

Invading bacteria are constantly arriving in the GI tract, but they must overcome the host’s tight defence and competition among microbes to achieve successful colonization. In this section, we summarize the key strategies by which invading bacteria break through defences and achieve successful colonization in the gut.

## Adhesion

**Figure 4.**
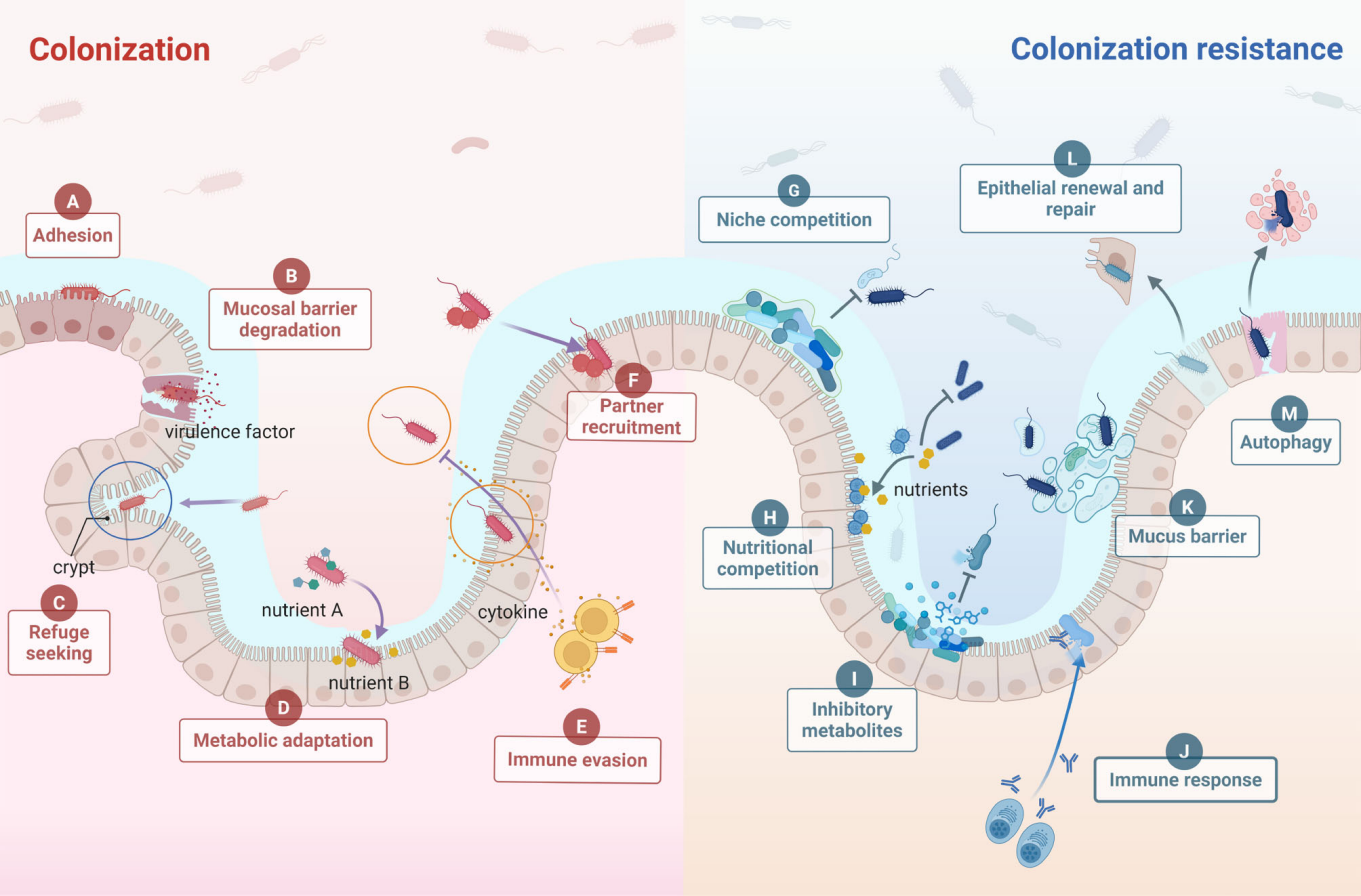
Colonization and colonization resistance mechanisms of the gut microbiota. Key strategies for gut colonization by foreign bacteria include (A) adhesion, (B) mucosal barrier degradation, (C) refuge seeking, (D) metabolic adaptation, (E) immune evasion, and (F) partner recruitment. Mechanisms of colonization resistance include (G) niche competition, (H) nutritional competition, (I) inhibitory metabolites, (J) immune response, (K) mucus barrier, (L) epithelial renewal and repair, and (M) autophagy. Created with BioRender.com.

When invading bacteria enter the GI tract, the critical initial step is adhesion. This process is mediated by bacterial surface structures, including outer membrane proteins, capsules, lectins, adhesins, and fimbriae (Liu et al. 2022, Jans et al. 2024). For example, pathogenic *E. coli* strains express various lectins with distinct sugar specificities, enabling binding to host mucins, glycoproteins, and extracellular matrix components (Donaldson et al. 2016). This adhesion system not only enables physical attachment but also triggers critical signal sensing, creating favourable conditions for subsequent colonization (Fig. 4A).

## Destruction of the mucosal barrier

Concurrently, colonization involves direct or indirect disruption of the mucosal barrier. Notably, several invasive GI bacteria have developed specific pathogenic factors that interfere with mucin production to cross the mucus barrier. For example, *Shigella* EATA enzyme analogues degrade MUC2 mucin to compromise the colonic mucus layer (Sheikh et al. 2022) (Fig. 4B).

## Ecological refuges

Certain invading bacteria exploit the anatomical structure and physiological characteristics of the GI tract to establish protected colonization environments. For example, *Helicobacter pylori* forms a persistent bacterial reservoir in the gastric glands, continuously supplying the transient bacterial population in the superficial mucosal layer through stable clonal populations (Fung et al. 2019). Similarly, intestinal crypts (Huang et al. 2011) and transverse folds (Nava et al. 2011) also provide sheltered habitats. These ecological niche specialization strategies highlight the importance of spatial organization in promoting bacterial colonization stability (Fig. 4C).

## Metabolic adaptation

Metabolic competition in the GI tract drives evolutionary adaptations in invading bacteria. *Escherichia coli* provides a notable example of metabolic adaptation, where mutations in specific metabolic genes confer the ability to utilize butyrate, a short-chain fatty acid that is not originally present in the culture medium but is subsequently produced by *E. coli* during active growth in fresh medium (Katz et al. 2023). *Clostridioides difficile* adapts to antibiotic-induced dysbiosis by increasing sporulation efficiency and bile acid metabolism (Mullish and Allegretti 2021). These metabolic adaptations illustrate the evolutionary plasticity of invading bacteria (Fig. 4D).

## Immune evasion

Successful gut colonizers have evolved mechanisms to modulate host immune responses. The persistence of *Bacteroides fragilis* is promoted through polysaccharide A-mediated induction of regulatory T cells (Round and Mazmanian 2010). Similarly, exopolysaccharides from *Bifidobacterium breve* promote immune tolerance through the coordinated suppression of proinflammatory cytokine release and B-cell activation (Fanning et al. 2012). The balance between protective immunity and microbial tolerance could selectively increase protection against invading bacteria (Fig. 4E).

## Recruiting symbiotic partners

Colonization by invading bacteria in the GI tract is not isolated; these microbes frequently recruit other microorganisms, which can include both coinvading and already resident microbes, to establish stable communities. The enhanced virulence of *H. pylori* in the presence of gastric streptococci (Sung et al. 2020) and the metabolic synergy between *C. difficile* and *Enterococcus faecalis* (Smith et al. 2022) demonstrate the ecological flexibility and complexity of gut adaptation (Hu et al. 2025). These recruitment dynamics are highly context-dependent and influenced by factors such as the traits of the invader and the state of the resident community (Fig. 4F).

## Mechanisms of colonization resistance

The GI tract has evolved sophisticated multilayered defence systems that collectively establish colonization resistance against invading bacteria (Smith et al. 2023). Understanding these protective strategies provides crucial insights for developing microbiota-targeted therapies aimed at restoring gut microbial homeostasis.

## Niche competition

Under homeostasis, most available niches are occupied—a key determinant of colonization resistance (Smith et al. 2023). Niche preemption has priority effects: early colonizers deplete growth-limiting resources, inhibiting late-arriving competitors with overlapping niches (Woelfel et al. 2024). More precisely, local bacteria limit the survival and reproduction of invading species by occupying critical intestinal niches and exerting selective pressure on invading communities (Seedorf et al. 2014). *Bifidobacterium* and lactic acid bacteria share this mechanism by forming protective biofilms that competitively exclude invading bacteria from epithelial surfaces (Nicholson et al. 2012). These observations underscore how the previously established microbial community structure intrinsically contributes to colonization resistance (Fig. 4G).

## Nutritional competition

The commensal microbiota maintain colonization resistance through active nutrient competition, creating an environment unfavourable for invading bacterial growth (Wang et al. 2024). For example, the commensal *E. coli* strains HS and Nissle 1917 synergistically inhibit *E. coli* O157:H7 by collectively competing for essential mucosal sugars (Maltby et al. 2013). Metagenomic analyses revealed that microbial communities with overlapping nutritional profiles engage in intense substrate competition, where superior nutrient acquisition strategies determine ecological success (Levy and Borenstein 2013). This metabolic competition extends to micronutrients such as iron, where commensals sequester essential metals through high-affinity acquisition systems, effectively starving invading bacteria (Lyng et al. 2024) (Fig. 4H).

## Inhibitory metabolites

In addition to resource competition, symbiotic bacteria produce various antimicrobial compounds that directly inhibit the survival of invading bacteria (Woelfel et al. 2024). SCFAs, particularly butyrate, acetate, and propionate, demonstrate potent antibacterial effects against multiple pathogens, including drug-resistant *Enterobacterales*, by promoting intracellular acidification (Sorbara et al. 2019). These fatty acids also regulate mucosal immune functions through multiple cellular pathways and enhance the antimicrobial capacity of monocytes and macrophages while inducing anti-inflammatory and tolerance responses in lymphocytes (Mukhopadhya and Louis 2025). Overall, the metabolic-immune barrier mediated by SCFAs establishes a defence mechanism against invasive bacterial colonization (Mann et al. 2024) (Fig. 4I).

## Immune response

The immune system serves as the host’s primary defence by dynamically interacting with the gut microbiota, playing a crucial role in shaping microbial communities (Macdonald and Monteleone 2005). When harmful invading bacteria attempt to colonize, they are inhibited by the resident microbiota through immune cell activation (Macpherson and Uhr 2004). For example, segmented filamentous bacteria induce IL-22 production, which enhances resistance to *Citrobacter rodentium* infection. Additionally, microbiota-primed IL-1β production in macrophages accelerates inflammatory responses against *Salmonella* Typhimurium and *Pseudomonas aeruginosa* through pro-IL-1β conversion (Smith 2023). The gut epithelium itself can also directly sense commensal bacteria and invading bacteria, such as through mammalian pattern recognition receptors (PRRs). PRRs recognize conserved bacterial and viral structures and generally activate proinflammatory signalling cascades that initiate host defence responses against potential infections (Takeda and Akira 2004). Together, these coordinated mechanisms demonstrate the sophisticated immune‒microbiota crosstalk that maintains gut homeostasis (Fig. 4J).

## Mucus barrier

In a healthy human intestine, the mucus layer is usually several hundred microns thick (McCallum et al. 2024). It not only provides a habitat for beneficial microorganisms but also forms a physical barrier that prevents invading bacteria from contacting epithelial cells. The main components of the mucus layer in the GI tract are mucins, which are O-glycosylated glycoproteins; secreted MUC2 forms peptide crosslinks to create a viscous gel-like substance, which serves as a barrier and host defence mechanism (Donaldson et al. 2016). Notably, the maintenance of mucus barrier integrity relies on continuous stimulation from the gut microbiota. For example, beneficial strains such as *Lactobacillus plantarum* 299v can promote the production of the intestinal mucoproteins MUC2 and MUC3, effectively blocking invading bacteria such as *E. coli* from adhering to intestinal epithelial cells (Mack et al. 2003). In addition, the flow of mucus continuously renews the mucus layer, facilitating the elimination of bacteria and debris. This self-purification mechanism further strengthens the ability of the GI tract to resist invading bacterial colonization (Hansson 2019) (Fig. 4K).

## Epithelial cell renewal and repair

Intestinal stem cells drive rapid epithelial renewal and maintain the highly dynamic intestinal epithelial structure, with cells completing self-renewal every 4–7 days (Odenwald and Turner 2017). The self-renewal capacity of these cells not only ensures the integrity of the epithelial barrier but also contributes to its repair after injury (Malagola et al. 2024). When invading bacteria are present, the JAK-STAT and JNK signalling pathways are activated, which work together to promote intestinal stem cell proliferation and epithelial regeneration to prevent bacterium-induced damage (Buchon et al. 2009). In addition, the renewal and continuous shedding of intestinal epithelial cells physically removes attached bacteria (Fig. 4L).

## Epithelial cell autophagy

Autophagy is a conserved antibacterial defence mechanism that involves the transport of intracellular bacteria to lysosomes for degradation. When bacteria enter epithelial cells, they trigger autophagosome formation, which eliminates invading bacteria and prevents their spread (Li et al. 2024). For example, during pathogenic invasion, subsets of *Salmonella enterica* subsp. *enterica* serovar Typhimurium (*S*. Typhimurium) enter cellular compartments to access the cytosol, where a portion of these intracellular bacteria become targets for autophagic recognition during the initial infection stages (Birmingham and Brumell 2006). Current research confirms the fundamental role of autophagy in the recognition and elimination of endocytosed bacteria and establishes this process as a fundamental component of the host’s innate defence (Benjamin et al. 2013) (Fig. 4M).

## Translating ecological mechanisms into therapeutic strategies

**Figure 5.**
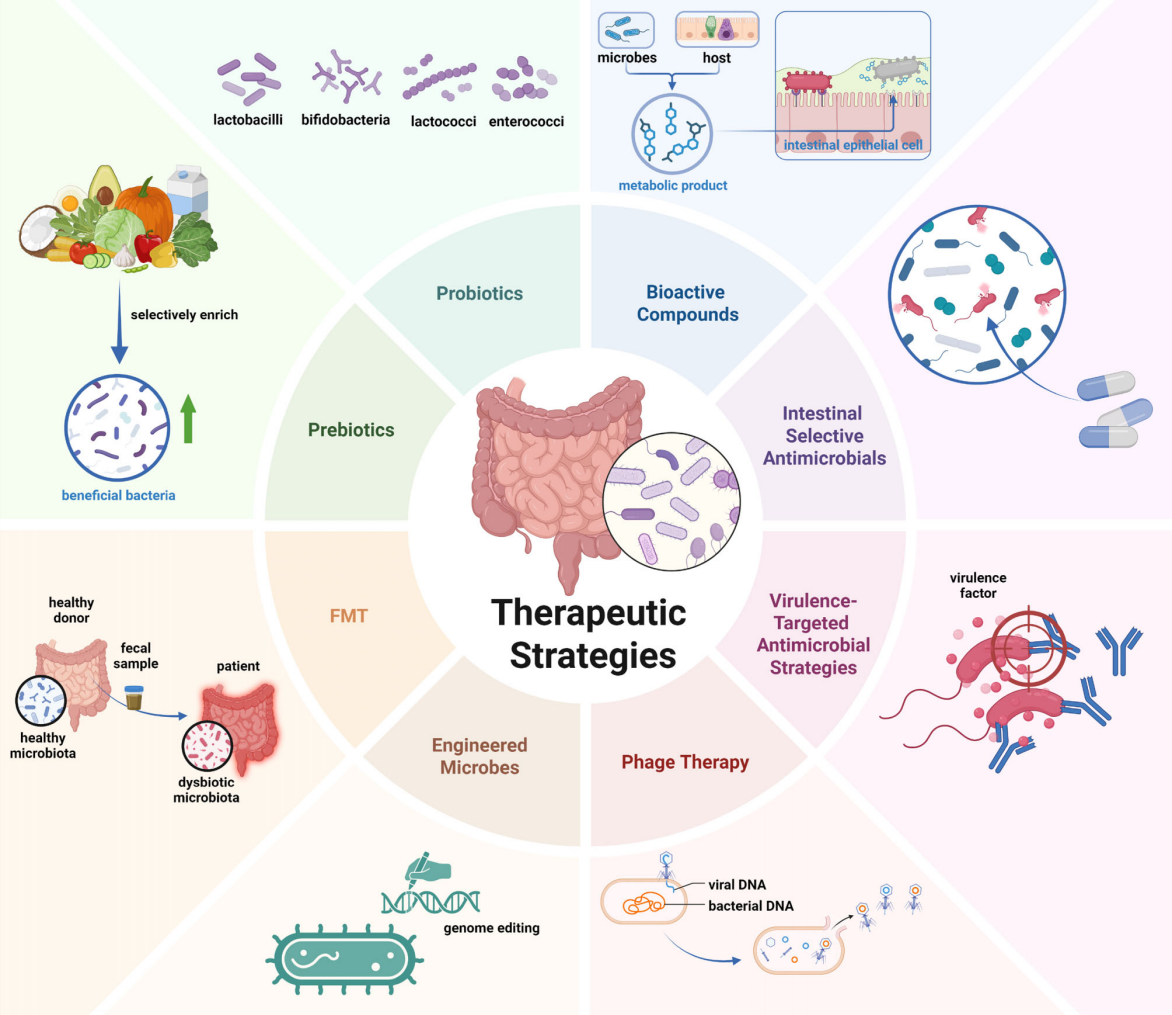
Multiple therapeutic strategies targeting colonization resistance. Eight potential therapeutic approaches to modulate colonization resistance and restore microbial balance, including prebiotics, probiotics, bioactive compounds, selective antimicrobials, virulence-targeted antimicrobials, phage therapy, engineered microbes, and faecal microbiota transplantation (FMT). Created with BioRender.com.

The complex interplay between host–microbe interactions and microbe‒microbe interactions has inspired multiple therapeutic strategies targeting colonization resistance. Recent advances have identified multiple approaches to modulate colonization resistance, ranging from holistic to precise antimicrobial strategies (Fig. 5).

## Prebiotics: dietary shaping of microbial metabolism

Prebiotics are substances that can be selectively utilized and transformed by the intestinal flora under the premise of improving host health. They can stimulate the proliferation and activity of gut microbes associated with health, thereby enhancing resistance to colonization by invading bacteria. Clinical evidence suggests that prebiotics such as inulin can selectively favour the growth of symbiotic bacteria such as *Bifidobacterium* (Riva et al. 2023) to increase colonization resistance against invading pathogens. In mouse models, inulin not only inhibited the growth of *C. difficile* (Hryckowian et al. 2018) but also enhanced the barrier function of the intestinal epithelium by increasing the levels of acetate, butyrate, and propionate (Corrêa et al. 2023). While the colon is a major site of prebiotic fermentation, emerging evidence reveals that inulin also has significant effects on the small intestine. Recent research has demonstrated that inulin intake enhances SCFA production specifically through the small intestine microbiome, which may provide a more immediate modulation of the gut environment and host metabolism (Jung et al. 2025). These findings suggest that prebiotics can selectively enrich beneficial bacteria to increase their resistance to colonization.

## Probiotic strains: reinforcing commensal defence networks

Probiotics are composed of live microorganisms that can benefit the health of the host when they are consumed in sufficient quantities (Mazziotta et al. 2023). Probiotics can competitively inhibit the binding of pathogens to intestinal mucosal receptors and release inhibitory metabolites to maintain the balance of the intestinal microbiota (van Zyl et al. 2020). Most probiotics, however, are only transient visitors in the GI tract and have difficulty achieving permanent colonization. They can achieve longer-term colonization by recruiting and collaborating with compatible microbial partners. Crucially, the success of this strategy often depends on the pre-existence of these compatible partners within the recipient’s indigenous gut microbiota. If such partners are absent or scarce, the recipient’s ecosystem may present stronger resistance to colonization, thereby limiting the persistence of the probiotic (Schmidt et al. 2018). This variability in individual microbial backgrounds may partly explain the heterogeneous colonization outcomes observed in response to probiotic interventions. A representative example is *Leuconostoc citreum*, which promotes the intestinal colonization of *Lactiplantibacillus plantarum* by collaborating with *Lactiplantibacillus plantarum* to reshape the intestinal microenvironment (Jiang et al. 2024). These findings suggest that microbial cooperation is important for maintaining the long-term health benefits of probiotics and highlight the potential of administering defined microbial consortia, rather than single strains.

## Metabolic intervention: microbially derived bioactive compounds

Metabolites from both microbes and the host regulate interactions between the host and the gut microbiota, including SCFAs, amino acids, bile acids, and polysaccharides (Mukhopadhya et al. 2025). Butyrate serves as a key regulatory metabolite that alters the histone status at the MUC2 promoter in a dose-dependent manner, which has implications for epithelial protection (Burger-van Paassen et al. 2009). These findings were also confirmed in mouse experiments, in which butyrate administration to the rectum significantly increased the synthesis of mucin 2, indicating its importance in strengthening the intestinal mucus layer (Gaudier et al. 2009). These findings highlight the therapeutic potential of butyrate. However, translating such bioactive compounds into practical therapies, particularly via oral administration, faces considerable challenges for oral administration, primarily the instability of metabolites during transit through the upper GI tract and the difficulty in achieving targeted delivery to the large intestine.

## Rifaximin: intestinal-selective antimicrobial

Traditional broad-spectrum antibiotics often disrupt gut microbiota diversity (Ianiro et al. 2016), whereas the unabsorbed antibiotic rifaximin exhibits selective antibacterial properties (Ponziani et al. 2015). When used for the treatment of hepatic encephalopathy, rifaximin specifically targets pathogenic bacterial groups such as *Enterobacterales* and *Veillonella* while promoting the production of SCFAs (Li et al. 2021) without significantly altering overall microbial diversity levels (Ponziani et al. 2020). This localized antibacterial capability enables selective control over pathogenic bacteria (Airola et al. 2023).

## Precision pathoblockers: virulence-targeted antimicrobial strategies

The nonselective effects of antibiotics disrupt the gut microbiota balance, a phenomenon commonly termed ‘collateral damage’ (de Nies et al. 2023). To mitigate these adverse effects, novel ‘pathogen blockers’ have emerged as precision solutions that preserve microbial integrity while suppressing pathogenicity. For example, *H. pylori* flagellar motility inhibitors effectively prevent gastric colonization without altering the composition of the intestinal microbiota (Suerbaum et al. 2022). These targeted therapies mark a shift from traditional bactericidal drugs to precise interventions (Smith 2023).

## Phage therapy: pathogen-specific depletion

Phage therapy inhibits pathogen dissemination by targeting bacterial migrants. Through targeted binding to bacterial receptors, phage therapy achieves strain-level recognition within bacterial populations (Bertozzi Silva et al. 2016) while minimizing interference with symbiotic microbial communities. For example, the *E. coli* phage øPNJ-6 was shown to protect the murine gut from *E. coli* invasion by enhancing GI persistence and antimicrobial effects (Wu et al. 2024). Emerging strategies employ synergistic combination therapies, such as bacteriophage‒antibiotic combinations. When bacteria develop resistance to an antibiotic, phage therapy can restore their sensitivity to other antibiotics (Canfield et al. 2023). Although clinical trials involving phage therapy are still in their infancy, preclinical studies on its feasibility are rapidly developing (Airola et al. 2023).

## Engineered microbes: synthetic microbial therapeutics

Intestinal microbes can be genetically modified to treat microbial imbalances. Combining optogenetics and genetic engineering, scientists developed light-responsive *E. coli* Nissle 1917 strains embedded in upconversion microgels to achieve controlled gut colonization. In a DSS-induced colitis model, this system effectively reduced inflammation (Cui et al. 2021). This successful application exemplifies how synthetic biology provides innovative methods that not only deepen the basic understanding of the interaction between the host and microbiota but also provide new ideas for the targeted engineering of the intestinal microbial ecosystem (Arnold et al. 2016).

## Faecal microbiota transplantation: microbial community reconstitution

Although probiotic and prebiotic treatments have demonstrated efficacy in modulating the gut microbiome, faecal microbiota transplantation (FMT) currently represents the most promising strategy for substantially reconstituting the gut microbial community (Ghani et al. 2024). The greatest clinical success of FMT has been observed in the treatment of recurrent *C. difficile* infection (CDI) (Wang et al. 2022). FMT works through a variety of synergistic mechanisms, including competitively excluding invading bacteria by occupying ecological niches and reconstructing metabolic networks that produce antimicrobial compounds. In addition, the emergence of segmented transplantation of exogenous microbiota revealed differences in the spatial distribution of GI colonization and highlighted the importance of ecological niche adaptation for successful transplantation (Li et al. 2020). However, FMT still faces many challenges, including the selection of donor microbiota, the safety of microbial transplantation, and the identification of biomarkers for key mechanisms (Schmidt et al. 2022). Solving these problems will advance FMT from empirical intervention to ecologically driven precision medicine.

## Challenges and prospects

Intestinal microbiota colonization resistance stems from the multilevel interactions between the host and microbes and between microbes themselves. In a stable state, the resident symbiotic microbiota establishes a formidable barrier against invaders through strategies such as nutrient competition, spatial dominance, and metabolic suppression (Woelfel 2024). Crucially, this environment of intense competition exerts strong selective pressure, driving bacteria to evolve sophisticated adaptive strategies. These strategies often involve a mix of both cooperative and competitive interactions, collectively fueling a complex and dynamic process of ecological evolution in the gut (Sulaiman et al. 2025).

By looking at the intestinal ecosystem as a whole and considering both individual and overall perspectives, studying the microbiota and intervention strategies in a comprehensive and systematic way will increase effectiveness and safety (Radlinski and Bäumler 2023). Experimental studies can start with a single strain but should ultimately evaluate the microbiota as a whole to study the overall trends and balance of microbial symbionts (Donaldson 2016). In this context, in vitro gut models serve as a crucial intermediary bridge between reductionist single-strain studies and complex in vivo human trials. These models include Transwell systems, gut-on-chip, and 3D-bioprinted organoids (Bernal et al. 2022). The integration of organoids with organ chips is particularly promising for modelling diseases such as IBD (Macedo et al. 2023).

However, the complexity of the gut microbiome and individual differences pose significant challenges. These differences span microbial composition, host genetics, and immune/metabolic profiles, compounded by a lack of standardization in experimental models. To overcome these obstacles, integrating multiomics into a unified research framework is necessary (Yang et al. 2025). In silico models can integrate multiomics data from diverse populations to simulate and predict personalized microbial dynamics and host outcomes, thereby mapping the landscape of variability (Thiele et al. 2020). Moreover, deepening the mechanistic understanding of how specific microbial metabolites and functions directly interact with host immune and metabolic pathways is essential to move beyond correlations and establish causality (Basic et al. 2022).

The intricate interactions between the human microbiome and the host organism exemplify the sophistication of biological regulatory systems. The complexity of the gut microbiota demands multidimensional therapeutic approaches. While emerging technologies such as genetically modified probiotics and phage therapy show promise for personalized microbial regulation, their clinical applications still require further validation to establish treatment protocols and efficacy standards (Airola 2023). The combination of probiotics, bacteriophage preparations, and FMT to construct a complete ‘antibacterial-repair-regulation’ therapeutic chain may be a promising future direction.

## Data Availability

Data sharing is not applicable to this article as no new data were created or analysed in this study.
